# Domain 3a mutation of VPS33A suppresses larval arrest phenotype in the loss of VPS45 in *Caenorhabditis elegans*

**DOI:** 10.17912/micropub.biology.001155

**Published:** 2024-03-21

**Authors:** Keiko Gengyo-Ando, Masahiko Kumagai, Hideki Ando, Junichi Nakai

**Affiliations:** 1 Oral Physiology, Tohoku University Graduate School of Dentistry, Miyagi, Japan; 2 Bioinformatics Unit, Research Center for Advanced Analysis, National Agriculture and Food Research Organization, Tsukuba, Ibaraki, Japan

## Abstract

The Sec1/Munc18 (SM) protein VPS45 is a key regulator of SNARE-mediated membrane fusion in endosomal trafficking, but its precise role remains unknown. To understand the function of VPS45 *in vivo* , we performed a genetic suppressor screen in *Caenorhabditis elegans* . We found that the temperature-sensitive lethality caused by the loss of VPS-45 can be suppressed by a mutation in another SM protein, VPS33A. The VPS33A M376I mutation is located in domain 3a, which is predicted to be essential for SNARE complex assembly. These results highlight the functional importance of domain 3a in endosomal SM proteins and its role in specific membrane fusion.

**Figure 1. vps-33.1 mutations suppress vps-45(0) ts larval arrest and lethality:**
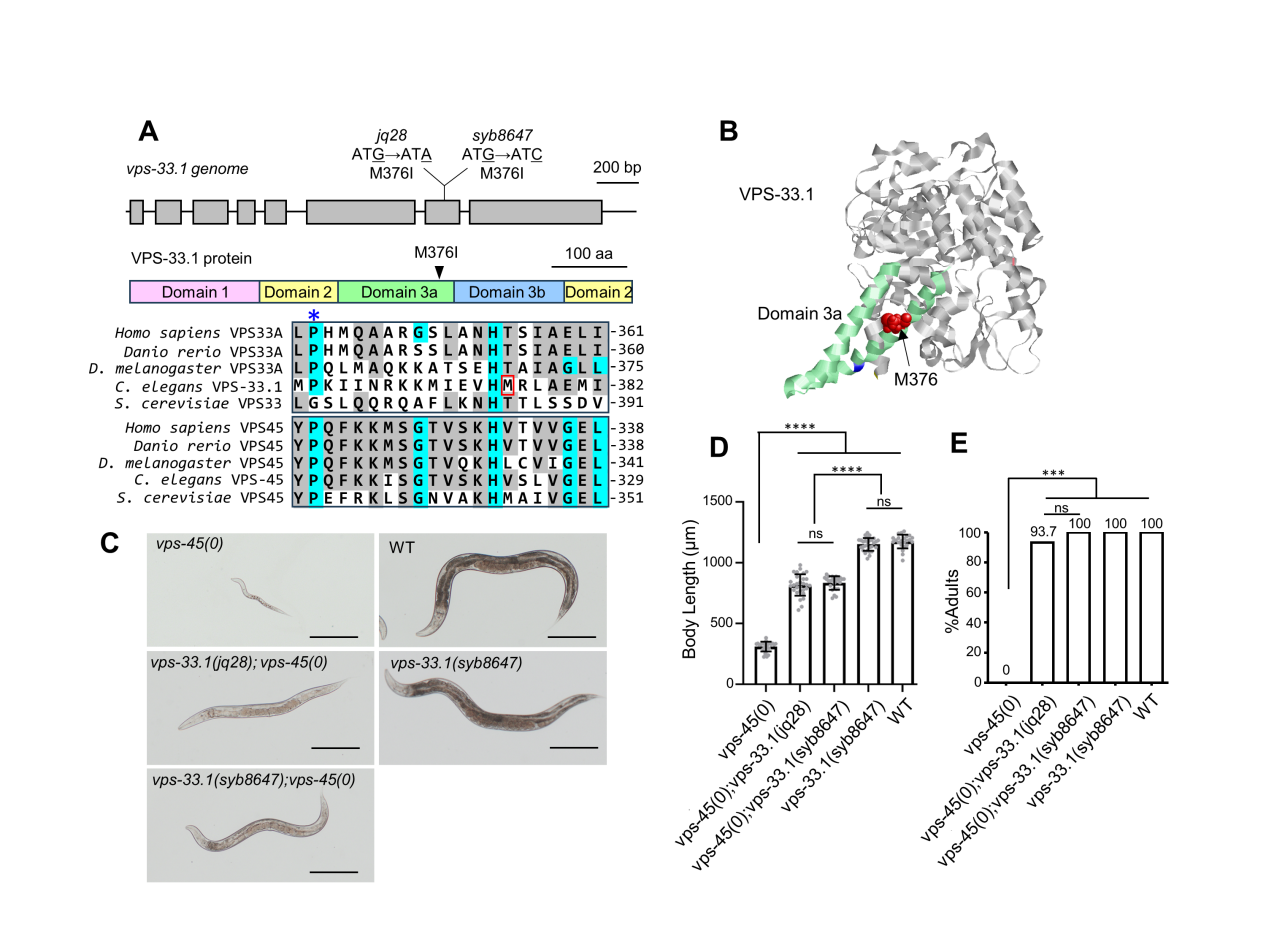
(A) Genomic region of the *vps-33.1* gene. Exons are indicated by gray boxes, mutation sites in *jq28* and *syb8647* are underlined, and amino acid substitutions are indicated below. The VPS-33.1 proteins contain three domains, with domain 3 split into domain 3a and domain 3b. The suppressor mutation associated residue methionine 376 (M376I, arrowhead) is located in the domain 3a. The amino acid sequence alignments of VPS33A and VPS45 are shown below [VPS33A: *Homo sapiens* (NP_075067.2), *Danio rerio* (NP_001093443.1), *Drosophila melanogaster* (AAF48972), *Caenorhabditis elegans* (CCD61712.1), *Saccharomyces cerevisiae* (KZV09647.1), VPS45: *Homo sapiens* (NP_009190), *Danio rerio* (NP_001243585.1), *Drosophila melanogaster* (AAF54403.1), *Caenorhabditis elegans* (NP_741714.1), *Saccharomyces cerevisiae* (CAA96801.1)]. The conserved amino acids of the VPS33 or VPS45 proteins are highlighted in grey and the conserved amino acids of both proteins are highlighted in light blue. The M376 is indicated by a red box. The strictly conserved proline (proline 363 in *C. elegans* ) in the domain 3a (Hu *et al.* , 2011) is indicated by a blue asterisk. (B) Structure of the *C. elegans* VPS-33.1 protein based on I-TASSER structural modeling. The 3D model with the highest confidence score is shown. The distal tip of domain 3a which is expected to interact with SNAREs/SNARE complex (Pieren, Schmidt and Mayer, 2010; Hu *et al.* , 2011; Baker, Jeffrey and Hughson, 2013) is shown in green. The residue M376 is indicated by a space-filling representation in red. The conserved proline, P363, is highlighted in blue. (C) Representative images of wild-type, *vps-45 (0)* , *vps-33.1 ( jq28 ); vps-45 (0)* , *vps-33.1 ( syb8647 ); vps-45 (0)* , *vps-33.1 ( syb8647 )* animals grown from eggs at 25°C for 76 h. *vps-45 (0)* represents the null allele *vps-45 ( tm246 )* . Scale bars, 200 µm. (D) Quantification of body length of animals in (C) (n=23-29). mean±SD. Statistics: one-way ANOVA with Dunnet's post hoc test. ****p<0.0001. ns: not significant. (E) Percentage of adults in the animals measured in (D) (n=23-29). ***p<0.001. ns: not significant. Statistics: Fisher's exact test, two-tailed.

## Description

Endosomal trafficking is an essential cellular process for cell organization and cell-cell communication. The Sec1/Munc18 (SM) family is a key regulator of Soluble N-ethylmaleimide–sensitive factor attachment protein receptors (SNAREs) and contribute to ensure the specificity of membrane fusion. In metazoans, three SM proteins, VPS33A, VPS33B and VPS45, are known to function in endosomal trafficking, but the functional differences and overlap between endosomal SM proteins are not fully understood.

The SM protein VPS45 regulates early endosome fusion and recycling (Nielsen et al *.* , 2000; Gengyo-Ando et al., 2007; Morrison et al., 2008; Scheidel, Kennedy and Blacque, 2018). Complete loss of VPS45 results in embryonic lethality in mice, and missense mutations in VPS45 cause immunological disease in humans (Frey, 2021). In this study, we performed a genetic suppressor screen in *C. elegans* to understand the functions of VPS45 *in vivo* . The *C. elegans* VPS45 homolog, VPS-45 , shares a high degree of sequence homology with human VPS45 (47% identity and 65% similarity) (WormBase version WS291; Davis et al., 2022), and loss of VPS-45 results in temperature-sensitive ( *ts* ) lethality and endocytosis defects. A null allele *tm246* (hereafter referred to as *vps-45 (0)* ), which deletes exons 1-4 in the *vps-45* gene on chromosome X, grows slowly to adulthood at a permissive temperature (15℃), but causes developmental arrests at the early larval stages at a restrictive temperature (25℃). To isolate suppressor mutants of *vps-45* , we mutagenized *vps-45 (0)* mutant animals with ethyl methanesulfonate (EMS) at 15℃, split F1s into separate populations, and then subjected the F2 offspring to 25℃. By screening approximately 90,000 haploid genomes, we isolated five independent suppressor strains that grow at the restrictive temperature. Here, we report one of these suppressor alleles, *jq28* .

*jq28* allele was genetically behaved as a dominant suppressor for *vps-45 (0).* By single nucleotide polymorphism (SNP) mapping, *jq28* mutation was mapped to the center of chromosome III (WormBase version WS291; Davis et al., 2022), and further analyzed by whole-genome sequencing (WGS). Based on the mapping data and WGS analysis, we found a candidate mutation (AT G to AT A , M376I; methionine at position 376 was replaced with isoleucine) in the coding sequence of the *C. elegans* VPS33A homologue ( *vps-33.1* ) ( Figure 1A ). To validate the causative suppressor mutation, we generated a *vps-33.1* allele, *syb8647* , carrying a mutation causing the identical amino acid substitution (AT G to AT C , M376I) by CRISPR-Cas9 gene editing ( Figure 1A ). To test whether the M376I mutation of VPS-33.1 is sufficient to suppress the *ts* lethality in *vps-45 (0)* , we generated *vps-33.1 ( syb8647 ); vps-45 (0)* double mutants and examined their development at the restrictive temperature. We found that the *vps-33.1 ( syb8647 ); vps-45 (0)* double mutant can grow to adulthood at the restrictive temperature at which the *vps-45 (0)* single mutant exhibits the larval arrest phenotype with complete penetrance ( Figure 1C -E). Under these conditions, the body length of the *vps-33.1 ( syb8647 ); vps-45 (0)* was almost the same as that of the *jq28* suppressor mutant, approximately 70% of that of the wild type ( Figure 1D ). These results indicate that the VPS-33.1 M376I is the causative mutation of the *vps-45 (0)* suppressor allele *jq28* .

Next, to investigate the structural basis of the *C. elegans* VPS33A, we performed an *in silico* structure prediction of VPS-33.1 using the I-TASSER server (Roy, Kucukural and Zhang, 2010) . VPS-33.1 shares a conserved topology of SM proteins with three domains (domain 1, domain 2, domain 3a, and 3b) ( Figure 1B ). The suppressor mutation (M376I) is located in the helical hairpin of domain 3a ( Figure 1A and B ), which is predicted to be essential for SNARE complex assembly (Baker, Jeffrey and Hughson, 2013; Han et al., 2013; Baker et al., 2015) . These results suggest that in the absence of VPS45, VPS33A, when mutated in the domain 3a ( VPS-33.1 with the M376I mutation), compensate for the function of VPS45 in the endosomal fusion events. Indeed, the domain structure of VPS33A and VPS45 is similar, and the amino acid corresponding to methionine 376 (M376) in VPS-33.1 is valine 323 (V323) in VPS-45 ( Figure 1A ). Interestingly, in the suppressor mutant, M376 is replaced by isoleucine, which is a branched-chain amino acid (BCAA) like valine or leucine, and BCAAs (valine or leucine) are conserved in the corresponding amino acids of metazoan VPS45 proteins ( Figure 1A ).

The single mutant *vps-33.1 ( syb8647 )* grew normally comparable to the wild type ( Figure 1C and D ). *vps-33.1* null mutation results in an embryonic lethal phenotype (Gengyo-Ando et al., 2016), suggesting that the M376I mutation has little effect on the core function of VPS-33.1 . Furthermore, the M376I mutation can restore larval development in the *vps-45* mutant, suggesting that the M376I mutation in VPS-33.1 , with or without VPS-45 , does not inhibit larval development.

In the endolysosomal system, at least three multisubunit tethering complexes exist and function in membrane fusion. These complexes contain specific endosomal SM proteins; VPS-33.2 in the class C core vacuole/endosome tethering (CORVET) complex, VPS-33.1 in the homotypic fusion and protein sorting (HOPS) complex and VPS45 in the factors for endosome recycling and retromer interactions (FERARI) complex (Solinger and Spang, 2014; Solinger et al., 2020) . Recent studies have reported hybrid tethering complexes in mammalian cells in which one of the subunits of HOPS or CORVET is replaced by the subunit of the other (Terawaki et al., 2023) . Therefore, it is conceivable that in the absence of VPS-45 , a new hybrid tethering complex containing the VPS-33.1 M376I mutant could be formed and act in both the VPS-33.1 -dependent fusion event and the VPS-45 -dependent fusion event.

## Methods

***C. elegans* strains and maintenance:** *C. elegans* strains were cultured on Nematode Growth Medium (NGM) seeded with *E. coli* OP50 as described (Brenner, 1974) . The wild-type strains Bristol N2 and the Hawaiian CB4856 strains were obtained from the Caenorhabditis Genetics Center. Nematodes were grown at 20°C, unless otherwise noted. The temperature-sensitive strain *vps-45 ( tm246 )* was maintained at 15°C. Strains generated in this study are listed in Reagents.

**Genome editing:** PHX8647 *vps-33.1 ( syb8647 )* mutant was generated by SunyBiotech (https://www.sunybiotech.com) using CRISPR-Cas9-mediated genome editing (Paix et al., 2015) with the guide RNA: CCG AAATGATTCAATCACATGTG (PAM in underlined) and the single-stranded oligodeoxynucleotide ( *ssODN* ): TCAAGAAAATGCCAAAAATTATTAATCGAAAAAAGATGATTGAAGTGCACAT **C** CG *G* CTTGC *G* GAAATGATTCAATCACATGTGTACTGTAAGCAGTCGGATTCGATTAAATT (mutations are underlined; a missense mutation in bold, synonymous mutations in italics).

**Suppressor screen:** To isolate gene suppressors that exhibit suppression of the *ts* lethal phenotype of *vps-45 ( tm246 )* mutants, a forward genetic screen using ethyl methanesulfonate (EMS) mutagenesis was performed as previously described (Brenner, 1974) . L4 hermaphrodites (P0, parental generation) of *vps-45 ( tm246 )* were mutagenized with 50 mM EMS for 4 h at the permissive temperature (15°C). Approximately 45,000 F1 progeny (approximately 90,000 haploid genomes) were divided into separate populations and incubated at 25°C for one week. Viable F3-F4 progeny were selected as *vps-45 ( tm246 )* suppressors. Five stable lines derived from independent F1s were isolated, and one of these suppressor alleles, *jq28* , was further analyzed in this study. To determine whether *jq28* was either a dominant or recessive mutation, *vps-45 ( tm246 ); jq28* and *vps-45 ( tm246 )* were each crossed to N2 males at 15°C. The F1 cross progeny males ( *vps-45 /0; jq28 /+* ) were viable at 25°C, whereas *vps-45 /0* males showed a *ts* lethal phenotype. Thus, the *jq28* allele showed a genetically dominant suppression of *vps-45* *ts* lethality.

**Genetic mapping:** Genetic mapping of the suppressor mutation was performed using the Hawaiian CB4856 mapping strain as described (Davis et al., 2005; Hwang and Wang, 2021) . *jq28 ; vps-45 ( tm246 )* hermaphrodites (Bristol N2 background) were crossed to the Hawaiian CB4856 males at 25°C. F1 cross progeny males that are hemizygous for *tm246* and heterozygous for *jq28* were crossed to *vps-45 ( tm246 ) ^Hw^* hermaphrodites, which are *vps-45 ( tm246 )* outcrossed 6 times in the Hawaiian CB4856 background. Hermaphrodite F2 cross progeny that are homozygous for *tm246* and heterozygous for *jq28* were cloned onto individual plates and incubated at 25°C. Worm lysates from fifty F2 plates with surviving F3-F4 offspring, i.e. those with *jq28* , were used as templates in PCR reactions to amplify SNPs or insertion-deletion polymorphisms (indels) spanning all chromosomes. PCR amplicons were analyzed by 2% agarose gel electrophoresis or MultiNA (SHIMADZU, Kyoto, Japan).

**Whole genome sequencing:** Genomic DNA for whole genome sequencing was extracted using the NucleoSpin Tissue Kit (Takara, Japan). Whole genome sequencing of *jq28* was performed by AproScience (https://apro-s.com/) using their whole genome sequencing service with 30 Gb data output for 300x coverage of the genome by paired-end 150 bp sequencing on the Illumina HiSeqX platform. Quality control and adapter trimming were performed using trimmomatic 0.39 (Bolger, Lohse and Usadel, 2014) . Reads were then mapped to the reference genome (GCF_000002985.6) by using bwa-mem v0.7.17 (Li and Durbin, 2009) , and duplicated reads were removed using Picard v4.2.6.1 ( https://broadinstitute.github.io/picard/ ). GATK v4.2.6.1 (Van der Auwera and O'Connor, 2020) was used for variant calling, following the best practice workflow for germline short variant discovery. The variant effect on the gene function was estimated using snpEff v5.1d (Cingolani et al., 2012) . The resulting variant information was visualized using the Integrative Genomics Viewer (IGV) (Robinson et al., 2023) .

**Measurement of body length:** Synchronized adults were allowed to lay eggs on NGM plates at 15°C. After 5 h, adults were removed and the eggs were incubated at 25°C for 76 h. For body length measurement, animals were mounted on agarose pads and photographed using a Nikon microscope (ECRIPSE Ni, Nikon, 4x objective) equipped with a DP28 digital camera (Olympus). Quantification of body length was performed using cellSens software (version 1.7, Olympus). Animals (n=23-29) were measured for each genotype.

**Statistical procedures:** Statistical significance in the body length was tested using one-way ANOVA followed by Dunnett's test in GraphPad Prism 10.0.3 (GraphPad Software) ( Fig. 1D ). Differences in the percentage of adults were analyzed using a Fisher's exact test in Excel Tokei (Social Survey Research Information) ( Fig. 1E ).

**3D model:** Prediction of the 3D structure of *C. elegans* VPS-33.1 was performed using the I-TASSER server (https://zhanggroup.org/I-TASSER/) (Zhang, 2008; Roy, Kucukural and Zhang, 2010) . The domain structure of *C. elegans* VPS-33.1 was based on the three-domain structure of SM proteins defined by Misura et *al.* (Misura, Scheller and Weis, 2000) . The structure was visualized using RasMol (version 2.7.5.2) ( http://www.openrasmol.org/ ).

## Reagents

**Table:** 

Strain	Genotype	Source
QJ4138	*vps-33.1 ( jq28 )III; vps-45 ( tm246 )X *	This study
QJ4139	*vps-33.1 ( syb8647 )III; vps-45 ( tm246 )X *	This study
PHX8647	*vps-33.1 ( syb8647 )III *	SunyBiotech

## References

[R1] Baker Richard W., Jeffrey Philip D., Zick Michael, Phillips Ben P., Wickner William T., Hughson Frederick M. (2015). A direct role for the Sec1/Munc18-family protein Vps33 as a template for SNARE assembly. Science.

[R2] Baker Richard W., Jeffrey Philip D., Hughson Frederick M. (2013). Crystal Structures of the Sec1/Munc18 (SM) Protein Vps33, Alone and Bound to the Homotypic Fusion and Vacuolar Protein Sorting (HOPS) Subunit Vps16*. PLoS ONE.

[R3] Bolger Anthony M., Lohse Marc, Usadel Bjoern (2014). Trimmomatic: a flexible trimmer for Illumina sequence data. Bioinformatics.

[R4] Brenner Sydney (2003). Nature's Gift to Science (Nobel Lecture). ChemBioChem.

[R5] Cingolani Pablo, Platts Adrian, Wang Le Lily, Coon Melissa, Nguyen Tung, Wang Luan, Land Susan J., Lu Xiangyi, Ruden Douglas M. (2012). A program for annotating and predicting the effects of single nucleotide polymorphisms, SnpEff. Fly.

[R6] Davis M Wayne, Hammarlund Marc, Harrach Tracey, Hullett Patrick, Olsen Shawn, Jorgensen Erik M (2005). Rapid single nucleotide polymorphism mapping in C. elegans. BMC Genomics.

[R7] Davis P, Zarowiecki M, Arnaboldi V, Becerra A, Cain S, Chan J, Chen WJ, Cho J, da Veiga Beltrame E, Diamantakis S, Gao S, Grigoriadis D, Grove CA, Harris TW, Kishore R, Le T, Lee RYN, Luypaert M, Müller HM, Nakamura C, Nuin P, Paulini M, Quinton-Tulloch M, Raciti D, Rodgers FH, Russell M, Schindelman G, Singh A, Stickland T, Van Auken K, Wang Q, Williams G, Wright AJ, Yook K, Berriman M, Howe KL, Schedl T, Stein L, Sternberg PW (2022). WormBase in 2022-data, processes, and tools for analyzing Caenorhabditis elegans.. Genetics.

[R8] Gengyo‐Ando Keiko, Kuroyanagi Hidehito, Kobayashi Tetsuo, Murate Motohide, Fujimoto Kazushi, Okabe Shigeo, Mitani Shohei (2007). The SM protein VPS‐45 is required for RAB‐5‐dependent endocytic transport in
*Caenorhabditis elegans*. EMBO reports.

[R9] Gengyo‐Ando Keiko, Kage‐Nakadai Eriko, Yoshina Sawako, Otori Muneyoshi, Kagawa‐Nagamura Yuko, Nakai Junichi, Mitani Shohei (2016). Distinct roles of the two
VPS33
proteins in the endolysosomal system in
*Caenorhabditis elegans*. Traffic.

[R10] Han Gayoung Anna, Bin Na-Ryum, Kang Soo-Young Ann, Han Liping, Sugita Shuzo (2013). The domain-3a of Munc18-1 plays a crucial role at the priming stage of exocytosis. Journal of Cell Science.

[R11] Hu Shu-Hong, Christie Michelle P., Saez Natalie J., Latham Catherine F., Jarrott Russell, Lua Linda H. L., Collins Brett M., Martin Jennifer L. (2010). Possible roles for Munc18-1 domain 3a and Syntaxin1 N-peptide and C-terminal anchor in SNARE complex formation. Proceedings of the National Academy of Sciences.

[R12] Hwang Ho-Yon, Wang Jiou (2021). Fast genetic mapping using insertion-deletion polymorphisms in Caenorhabditis elegans. Scientific Reports.

[R13] Misura Kira M. S., Scheller Richard H., Weis William I. (2000). Three-dimensional structure of the neuronal-Sec1–syntaxin 1a complex. Nature.

[R14] Morrison Holly A., Dionne Heather, Rusten Tor Erik, Brech Andreas, Fisher William W., Pfeiffer Barret D., Celniker Susan E., Stenmark Harald, Bilder David (2008). Regulation of Early Endosomal Entry by the
*Drosophila*
Tumor Suppressors Rabenosyn and Vps45. Molecular Biology of the Cell.

[R15] Nielsen Erik, Christoforidis Savvas, Uttenweiler-Joseph Sandrine, Miaczynska Marta, Dewitte Frederique, Wilm Matthias, Hoflack Bernard, Zerial Marino (2000). Rabenosyn-5, a Novel Rab5 Effector, Is Complexed with Hvps45 and Recruited to Endosomes through a Fyve Finger Domain. The Journal of Cell Biology.

[R16] Paix Alexandre, Folkmann Andrew, Rasoloson Dominique, Seydoux Geraldine (2015). High Efficiency, Homology-Directed Genome Editing in
*Caenorhabditis elegans*
Using CRISPR-Cas9 Ribonucleoprotein Complexes. Genetics.

[R17] Pieren Michel, Schmidt Andrea, Mayer Andreas (2010). The SM protein Vps33 and the t-SNARE Habc domain promote fusion pore opening. Nature Structural & Molecular Biology.

[R18] Robinson James T, Thorvaldsdottir Helga, Turner Douglass, Mesirov Jill P (2022). igv.js: an embeddable JavaScript implementation of the Integrative Genomics Viewer (IGV). Bioinformatics.

[R19] Roy Ambrish, Kucukural Alper, Zhang Yang (2010). I-TASSER: a unified platform for automated protein structure and function prediction. Nature Protocols.

[R20] Scheidel Noémie, Kennedy Julie, Blacque Oliver E (2018). Endosome maturation factors Rabenosyn‐5/VPS45 and caveolin‐1 regulate ciliary membrane and polycystin‐2 homeostasis. The EMBO Journal.

[R21] Solinger Jachen A., Rashid Harun-Or, Prescianotto-Baschong Cristina, Spang Anne (2020). FERARI is required for Rab11-dependent endocytic recycling. Nature Cell Biology.

[R22] Terawaki Seigo, Vasilev Filipp, Moriwaki Takahito, Otomo Takanobu (2023). HOPS, CORVET and newly-identified Hybrid tethering complexes contribute differentially towards multiple modes of endocytosis. Scientific Reports.

[R23] Zhang Yang (2008). I-TASSER server for protein 3D structure prediction. BMC Bioinformatics.

[R24] Van der Auwera, G.A. and O’Connor, B.D. (2020) Genomics in the Cloud: Using Docker, GATK, and WDL in Terra. 1st Edition. O’Reilly Media.

[R25] Solinger Jachen A., Spang Anne (2014). Loss of the Sec1/Munc18-family proteins VPS-33.2 and VPS-33.1 bypasses a block in endosome maturation in
*Caenorhabditis elegans*. Molecular Biology of the Cell.

[R26] Li Heng, Durbin Richard (2009). Fast and accurate short read alignment with Burrows–Wheeler transform. Bioinformatics.

